# When three’s a crowd: how relational structure and social history shape organizational codes in triads

**DOI:** 10.1186/s41469-020-00078-9

**Published:** 2020-08-28

**Authors:** Özgecan Koçak, Massimo Warglien

**Affiliations:** 1 Goizueta Business School, Emory University, 1300 Clifton Road, Atlanta, GA 30322 USA; 2 Department of Management, Università Ca’ Foscari, Dorsoduro 3246, 30123 Venice, Italy

**Keywords:** Communication codes, Code convergence, Organizational structure, Conceptual pacts, Common ground, Coordination games, Experiment, Triads, Acyclicity, Transitivity, Hierarchy, Language

## Abstract

When members of an organization share communication codes, coordination across subunits is easier. But if groups interact separately, they will each develop a specialized code. This paper asks: Can organizations shape how people interact in order to create shared communication codes? What kinds of design interventions in communication structures and systems are useful? In laboratory experiments on triads composed of dyads that solve distributed coordination problems, we examine the effect of three factors: transparency of communication (versus privacy), role differentiation, and the subjects’ social history. We find that these factors impact the harmonization of dyadic codes into triadic codes, shaping the likelihood that groups develop group-level codes, converge on a single group-level code, and compress the group-level code into a single word. Groups with transparent communication develop more effective codes, while acyclic triads composed of strangers are more likely to use multiple dyadic codes, which are less efficient than group-level codes. Groups of strangers put into acyclic configurations appear to have more difficulty establishing “ground rules”—that is, the “behavioral common ground” necessary to navigate acyclic structures. These coordination problems are transient—groups of different structures end up with the same average communication performance if given sufficient time. However, lasting differences in the code that is generated remain.

## Introduction

Organizations often rely on idiosyncratic “codes” in their internal communication (Arrow 1974). Specialized lexica, tailored to their activities and coordination needs, allow more efficient communication than ordinary natural language. These lexica are important for coordination through verbal communication, and they play a critical role in building shared understandings such as implicit contracts and cultures (Weber and Camerer 2003; Gibbons and Henderson 2012; Srivastava and Goldberg 2017). A long-standing question, therefore, is how organizations can design effective communication codes that are shared across their members and subunits (Cremer et al. 2007).

This goal may be elusive, however, because typically codes are not designed, but emerge as solutions to coordination problems (Wernerfelt 2004). An optimally designed code would involve everyone in the organization attaching the same signifiers to the same signified, but “dialects” may emerge across subunits as a result of the trade-offs between overall coordination and the advantages of locally specialized communication. Indeed, students of linguistic interaction have shown that communication codes emerge through an interactive process of *grounding*, or the construction of a common ground of mutual beliefs, presuppositions, and mutually recognized background information among agents that participate in a joint activity (Stalnaker 1973; Clark and Wilkes-Gibbs 1986; Wilkes-Gibbs and Clark 1992; Brennan and Clark 1996; Clark 1996). Furthermore, since natural language is intrinsically underdetermined, its interpretation is shaped by the dynamics of conversations (Ludlow 2014). The code that emerges therefore depends on the history of such interactions. Even dyads that are left to develop independently specialized codes in the laboratory experience loss of coordination and efficiency in communication when combined into triads (Wilkes-Gibbs and Clark 1992; Galantucci et al. 2012). In groups that are created by integrating preexisting groups, who find themselves with conflicting codes, we see the same loss (Weber and Camerer 2003). This tendency is borne out by field studies, which show that organizations are riddled with codes that vary across subunits, sometimes causing coordination problems (Tushman and Katz 1980; Bechky 2003).

Prior work thus points to a dilemma: A common communication code facilitates coordination across groups. With an effective code, you can decentralize decision-making. Yet if groups interact separately, they will each develop a specialized code. This dilemma can be seen as an instance of the trade-off that all organizations face in dividing and integrating their members’ actions, which raises the possibility that it can be managed through organization design. Can organizations shape how people interact in order to create shared communication codes? What kinds of design interventions in communication structures and systems are useful? Can tools and technologies that are used to facilitate distributed work during the Covid-19 pandemic be useful after we are no longer required to work from home?

Interactions play a foundational role in code creation. This suggests that organizational structure may aid or hinder the construction of a unique shared organizational code in place of multiple local codes. Indeed, experiments show that dyadic codes can be harmonized and recreated at the group level by making everyone talk to everyone else (Garrod and Doherty 1994; Fay et al. 2010). Even in relatively large groups, appropriate patterns of interaction and sufficient levels of connectivity can ensure convergence on a common code (Centola and Baronchelli 2015). Computational studies support this finding, demonstrating that communication structures, defined in terms of who talks to whom, can be designed to support the emergence of a group-level convention out of dyadic interaction (e.g., Skyrms 2010; Barr 2004; Centola et al. 2018). At the same time, these studies suggest that the structure of grouping individuals (and interactions) into subunits can determine the dialects that will emerge in an organization.

However, social structures of communication are not limited to a specification of who talks to whom. This is evident even at the simplest level—combining dyadic relations into triadic ones, as observed by Simmel (1950: 136): “It is usual for just such finely tuned combinations of three at once to result in three parties of two persons each, and thus to destroy the unequivocal character of the relations between each two of them.” More broadly, organizations shape the course of conversations among their members in multiple ways (Gibson 2005). As sociologists have shown, the development of solutions to coordination problems is embedded in the nature of relations among agents (Granovetter 1985; Leifer 1988). The process of creating a common code in an organization is therefore likely to be shaped also by factors other than who talks to whom.

In this paper, we consider contextual factors that may facilitate the development of a shared code independent of connectivity. Psycholinguistic studies point to at least three factors that can impact code convergence and can be expected to vary across organizational situations. First, role differentiation in communicative interactions can impact how easy it is to converge on a common code (Selten and Warglien 2007). Organizational structures affect not only how often individuals interact, but also what role the individuals play within the interaction, such as who sends messages and who receives them, whether communication is symmetric or asymmetric, and whether individuals specialize in sending or receiving messages. Second, given that convergence on a code requires building common ground, prior common ground among agents makes it easier for them to develop a shared understanding and a new code (Clark 1996). Some organizational settings (such as new establishments, mergers, and cross-unit interactions) bring together agents who are strangers to one another, while others (such as stable units) feature repeated interactions among longtime colleagues, who can ground their current interaction more easily. Third, organizations can shape the formation of common ground by controlling how individuals access communication—that is, whether communicative interaction is private or transparently available to bystanders (Schober and Clark 1989).

Our empirical design aims at reproducing experimentally the emergence of codes in structured interactions between multiple agents, observing whether a common group-level code is generated and the nature of the code that is generated. We extend the experimental framework on dyadic matching games (Krauss and Glucksberg 1977; Clark and Wilkes-Gibbs 1986) to triads: we had groups of three individuals play dyadic matching games for which they were rewarded at the group level, based on speed and accuracy of communication. In each round of the game, a “receiver” was asked to find the target image (randomly drawn out of a set of 16 images) that the “sender” described. We recorded how fast dyads coordinated in each of the games they played, as well as each agent’s preferred label for each image at the end of the experiment (which consisted of 120 rounds for each triad). This setup allowed us to observe whether the groups developed any shared code—ranging from any label or description that all three people used to refer to the same image, to a single label or description that all three people preferred, to a single label that was a single word, with each of these codes more effective than the previous, as confirmed by our data on communication speed and accuracy. In addition to the frequency of communication among agents, which prior studies find to have an effect on likelihood of code convergence (e.g., Garrod and Doherty 1994; Fay et al. 2008, 2010; Centola and Baronchelli 2015), we examine the effect of the three aforementioned factors: transparency of communication, role differentiation, and the social history of the subjects (as observed by separating groups consisting entirely of strangers from groups including friends).

## Literature review

In this section, we briefly summarize the most relevant antecedents to our study and qualify how our experimental design is differentiated from them.

### Process of code emergence

Laboratory experiments on how communication codes and other linguistic conventions emerge go back to psycholinguistic studies that rely on dyadic coordination games (Krauss and Glucksberg 1977; Clark and Wilkes-Gibbs 1986). These studies found that dyads reliably developed common codes in relatively short periods of time, reaching an agreement on how linguistic expressions map to objects and events through a collaborative process of building common ground on shared knowledge, beliefs, and assumptions (Clark and Wilkes-Gibbs 1986; Brennan and Clark 1996; Clark 1996). More recent studies have extended the paradigm of studying emergence of codes among agents playing coordination games to a broader range of communication codes beyond natural language, including the creation of ex novo signs (Galantucci 2005), artificial languages (Selten and Warglien 2007; Kirby et al. 2008), and pictorial codes (Garrod et al. 2007). All these studies (see also Galantucci and Garrod 2011 for a review) confirmed earlier findings that dyadic communication can spontaneously generate stable shared codes, while qualifying conditions affecting the emergence process and its success.

The transition from dyadic codes to codes shared in larger groups of multiple dyadic interactions is not straightforward. While a specialized code increases the efficiency of communication between agents that share it, it has the opposite effect on their coordination with agents that are not socialized into it. In organizations, field research examples show such conflicts from divergent codes across different functional groups (Bechky 2003) and following mergers (de Vecchi 2012). Harmonization of dyadic codes is problematic even in the laboratory. Dyads that are left to develop independently specialized codes experience loss of coordination and efficiency in communication when combined into triads or larger groups. Weber and Camerer (2003) use this to illustrate difficulty of integration after mergers. Galantucci et al. (2012) show that most triads cannot develop a common code. Specifically, a group-level code is less likely to emerge if a member of the group is not able to keep up with the code that the other dyad creates, due to lower ability, limited participation in the task, less cooperation on the part of the other players, or a combination of these factors (Galantucci et al. 2012).

Differently from former experimental studies, we do not consider the emergence of a shared code as an all-or-none alternative. We consider instead different degrees of sharing, ranging from no shared code, to partially shared overlapping codes, to fully shared ones. Field studies in organizations show that organizations can host a range of codes, from those shared across the entire organization, to multiple codes overlapping across sub-units, to islands of non-overlapping codes (corresponding to the familiar complaints that one hears in organizations about “existence of siloes”). When multiple codes exist across organizational units, multi-lingual translators can compensate for the absence of a shared language between project groups and their external audiences (Tushman and Katz 1980). Indeed, computational models show that feasibility of translation increases the likelihood that overlapping codes exist across units, preventing the emergence of a common code (Immorlica et al. 2007).

Accordingly, we start our analysis of the emergent codes in our experiment by testing whether the nature of the emergent codes is associated with effectiveness of linguistic coordination. We test whether the existence of a code shared at the group level is associated with more effective communication than the existence of dialects, whether a shared code is more effective if it consists of a single word, and whether length of labels affects speed of coordination. These tests constitute the first attempt at validating the commonly held assumption that shared codes are more effective than multiple (possibly overlapping) ones, even for decentralized communication tasks (Cremer et al. 2007).

Attending to the different kinds of codes that can emerge in group interaction also allows us to question the process of code emergence in greater detail: What can explain whether the process of code emergence leads to a harmonization of codes versus agents sticking to their own preferred codes, a situation of “three parties of two persons each”, or some other combination?

### Common ground

Psycholinguists have shown that communication requires both coordination of content and coordination of process (Clark and Brennan 1991). Understanding each other requires a common ground—a set of mutual beliefs, presuppositions, and mutually recognized background information that create the context within which utterances become intelligible (Stalnaker 1973; Clark and Wilkes-Gibbs 1986). Common ground is “the field on which a language game is played” (Stalnaker 2002:720). However, common ground is often defective—speakers may have different beliefs or presuppositions. Communicative interaction is thus a process through which common ground is updated and accumulated—a process often referred to as grounding (Clark and Brennan 1991). Grounding in a particular interaction and thus communication is easier for communicators who share prior common ground, thanks to common membership in groups or a personal relationship (Clark 1996).

We extend this argument by positing that prior common ground among agents also helps them develop a new code to refer to novel stimuli. Agents that have prior common ground are more likely to have a shared lexicon or cultural reservoir of semantic tools such as metaphors that they can draw on to describe novel images. However, common ground and grounding do not have just a semantic or pragmatic nature—they have a social dimension that affects agents’ shared background information, their understanding of the interaction context within which communication happens, and the negotiation of communication roles. The nature of communication between agents who have no prior interaction is likely to be very different from interactions between agents with a common history. Understanding how prior common ground affects emergence of codes is especially relevant for understanding communication in organizations, which constitute and can purposefully design the context of communication, including who interacts with whom, what roles and positions they occupy in the social structure, and what goals they pursue in communication.

Our study therefore examines the effects of the social history of participants on code emergence and coordination performance. Specifically, we test whether past interactions, which proxy the existence of prior common ground among participants, increase the likelihood that they develop shared labels for the novel images that they encounter in our experiment.

### Communication structure

Studies in diverse fields ranging from evolutionary biology to computer science have used computational experiments on simulated agents to examine how a code may spread throughout a social system (e.g., Skyrms 2010; Barr 2004; Centola et al. 2018). Assuming that dyads converge to a common code through interaction, they find that the topography of interaction among agents influences whether a common code at the group level emerges or whether pockets of different codes persist. Laboratory experiments confirm this finding. Fully mixed experimental groups, that is, groups where everyone interacts with everyone else, reliably develop codes shared by everyone, whereas isolated dyads develop and continue to use their own specialized codes (Garrod and Doherty 1994; Fay et al. 2008, 2010). In groups where not everyone interacts with everyone else, the topology of the network, determining who interacts with whom, impacts the likelihood that a group-level code emerges (Centola and Baronchelli 2015). These studies predict that in organizations, where formal and informal structures create islands of interaction, frequency of interaction among individuals is likely to impact code emergence.

Much less investigated is the effect of role differentiation. Prior research finds that dyads that develop asymmetric roles within an experiment are more likely to converge on a code by having one agent adopt the other’s labels (Selten and Warglien 2007). While the effect of asymmetry at the dyad level is known, the effect at the group level has not been studied. Both computational studies and experimental studies of code convergence in groups have typically treated dyadic ties as symmetric. In organizations, however, who speaks to whom is likely to vary by role. Communication structures in organizations are frequently differentiated, if only because roles require some people to transmit information to others. For example, nurses changing shifts may relay information after each shift, to the nurses in the next shift (Wolf 1989). Or, design engineers may explain their blueprints to prototype technicians and to assemblers while technicians send specifications to assemblers (Bechky 2003).

We therefore extend prior studies on the effect of communication structures by examining the effect of role differentiation. We model organizational structures through triads, which are the smallest unit that enable the creation of a differentiated system of roles. In recognition of the importance that Simmel has accorded to triads, prior work has named dyads embedded within triads “Simmelian ties” (Krackhardt 1998), and Simmelian ties that span organizational boundaries “Simmelian bridges” (Tortoriello and Krackhardt 2010). Tortoriello and Krackhardt (2010) find that ties that bridge organizational boundaries improve innovation performance only if they are embedded in cliques. They attribute this to greater stability of Simmelian bridges facilitating the formation of common language and shared understandings. Our study holds stability constant while comparing two triadic configurations to examine the effect of role differentiation.

**Fig. 1:**
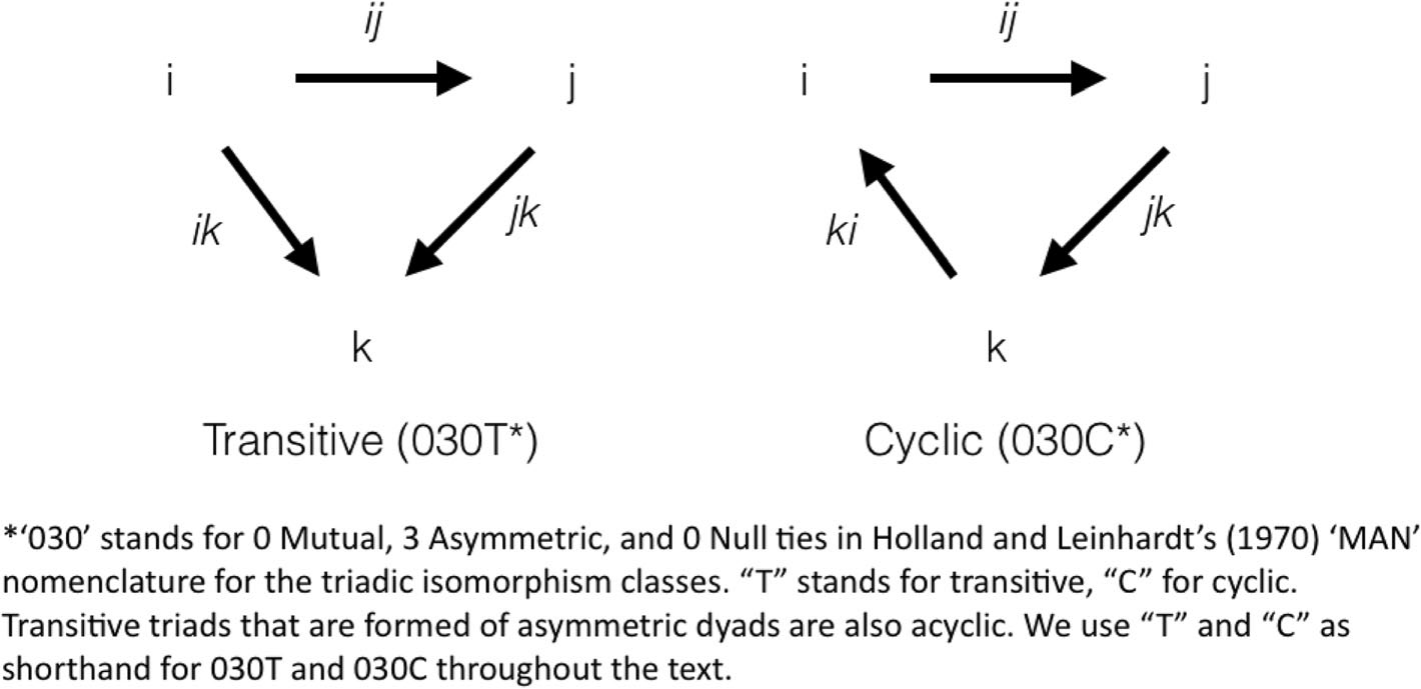
Triadic configurations studied

Three asymmetric dyads can be combined into a triad in two ways (Holland and Leinhardt 1970); Holland and Leinhardt 1976). One, that preserves transitivity of the asymmetric relationship, is illustrated on the left in Fig. 1. Isaac can give a report at the end of his nursing shift to nurses Jackie and Kendra while Jackie gives a report to Kendra. The acyclic combination of dyadic communications ij, ik, and jk create unique, differentiated positions for agents in the triad. Isaac only gives reports, Kendra only receives them, and Jackie does both. Acyclic transitive triads (labeled 030T according to Holland and Leinhardt’s nomenclature) are found more frequently in dominance hierarchies than would be expected on the basis of a dyadic census (Faust 2007). They are also seen in organization theory as a central characteristic of formal managerial structures (Bunderson et al. 2016) and informal leadership structures (Carnabuci et al. 2018). In a comparison of networks in two organizations, Tasselli and Caimo (2019) find that dyadic advice relationships across sub-units are more likely to exist within 030T configurations in the formal hierarchical organization. We therefore consider 030T triads to be a model for communication structures in hierarchical organizations. Acyclic triads need not only exist within hierarchies, however. For instance, the division of tasks within an engineering team may be such that *k* receives input on customer needs from both *i* and *j* while *j* only requires the input from *i*.

A useful comparison is the other triadic configuration that combines three asymmetric dyads. Illustrated on the right in Fig. 1, this configuration is cyclic (as reflected in its label “030C” given by Holland and Leinhardt). In this configuration, where Isaac gives a report to Jackie, Jackie to Kendra, and Kendra to Isaac, every individual and every dyad in the triad is structurally equivalent. In the study mentioned above, Tasselli and Caimo (2019) find boundary-spanning advice relationships to be embedded within 030C configurations in the less hierarchical organization. Faust (2007) also finds triads of this type to be less common than expected on the basis of a dyadic census, presumably because asymmetric dyads tend to combine into 030T rather than 030C. Note that the two configurations are identical except for one dyadic relationship “pointing in” the “opposite” direction.

Prior literature supports conflicting predictions for how role differentiation in groups may impact the nature of codes (net of the effect of interaction frequency). On the one hand, role differentiation has been shown to help information aggregation. Individuals placed in task groups of different communication structures find it easier to share information in structures that have a clear central position or at least some role differentiation (Leavitt 1951). Similarly, the acyclic structure can be expected to reinforce dyadic asymmetry, making it easier for dyads in transitive triads to converge on a code. In the cyclic structure, however, there is no positional difference to cue agents on how to coordinate on a common code. On the other hand, formation of conceptual pacts is a different problem than information aggregation given an existing common code. While it may be more efficient to diffuse ideas or information through a centralized network, creation of a common code is a coordination problem. Where multiple communicative relationships exist, differentiation may create more conflicts between emerging conceptual pacts. A differentiated structure may make it harder to create common ground on how to converge on a group-level code, especially when the order of dyadic interaction does not mirror the direction of differentiation. For instance, if Jackie and Kendra agree on a label for a particular image before Isaac ever sees the image and communicates it to Jackie or Kendra, the differentiated structure may do more harm than good. In other words, decentralized dyadic coordination within differentiated structures can disrupt development of group-level codes rather than facilitate convergence and harmonization.

At the same time, acyclic structures (such as transitive relations formed of asymmetric dyads) have been found to reduce conflict and enhance group performance in teams (Bunderson et al. 2016). Acyclicity can also presumably be useful for resolving conflict among dyadic codes. In the acyclic transitive triad, if Isaac and Jackie use different labels to refer to the same image, Kendra can prompt them to compromise on a common label. Or, Isaac, in reporting to both Jackie and Kendra, can make sure that he uses the same code with both. In the cyclic triad, however, the structure does not make it easier for any individual to play a coordinating role.

We examine the effect of role differentiation on code emergence by manipulating the micro-structure of communication. Specifically, we have our triads play coordination games in dyads that are either organized as in the acyclic configuration or the cyclic configuration in Fig. 1. All dyads in these triads are asymmetric. That is, our study controls for dyadic asymmetry by design. Each of the three dyads in a triad interacts with equal frequency on expectation, as the probability of each dyad being picked in any round is equal. Thus, our experimental conditions are also equivalent (in expectation) with respect to the degree of connectedness that has been studied in prior studies of communication structure. (In statistical analyses, we are able to control for the realized frequency of dyadic interaction.)

### Private versus transparent communication

Even in triads where everyone interacts with everyone else, one party in a dyad will not have direct experience of the other dyad’s interaction. This divisive aspect of structure can be overcome by making communication transparent, that is, by making dyadic interactions available to the third party. Privacy versus transparency of communication is a common choice that is made in organizations, for instance when one decides to open a communication to bystanders by copying them on e-mails or by using other collaborative technologies (Leonardi 2014). Prior studies in psycholinguistics finds that while bystanders do not internalize codes to the same extent that participants do, they nonetheless do better than third parties who were not privy to the communication (Schober and Clark 1989).

Studies that compare private versus transparent communication have not examined emergence of group-level codes (Clark and Schaefer 1989), while studies that have examined emergence of group-level codes have only featured transparent communication (Galantucci et al. 2012; Weber and Camerer 2003). One can expect that transparent communication will make common code emergence easier by facilitating grounding. Bystanders can learn how the other dyad labeled an object and use that knowledge when it is their turn to communicate. We test this conjecture by comparing the two types of access to communication.

We also test if transparent communication reduces any difference that exists between acyclic and cyclic groups or between groups with and without prior common ground. While we expect transparent communication to facilitate lexical grounding, its effect on bridging gaps in behavioral common ground may be more limited, unless individuals use the opportunity of transparent communication to establish “ground rules” for how to resolve conflicts between emerging codes.

## The experiment

The experiment consisted of matching games played by three dyads in a triad. In matching games, an individual in the role of “message sender” describes one image out of many to another, the “message receiver,” who tries to find the correct image. In order to examine how role differentiation influences the emergence of code, we created groups in one of two triadic configurations depicted in Fig. 1 (where the direction of arrows shows the message sender describing images to message receiver). The first configuration is characterized by acyclic and transitive relations, with the three agents in different relational positions (henceforth, “T”). Specifically, agent *i* always sends messages, agent *j* receives messages from *i* and sends messages to *k*, and agent *k* always receives messages in this configuration. The second is a cyclic configuration in which all agents have the same relational position (henceforth, “C”), sending messages to one partner and receiving messages from the other partner.

**Fig. 2:**
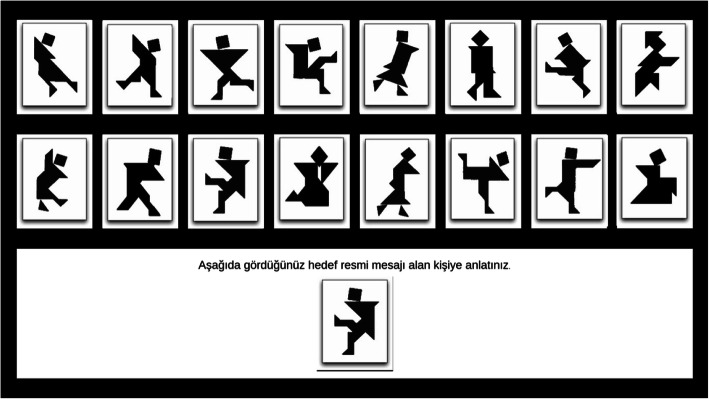
Message sender’s screen: Instructions indicate that selected target image should be conveyed to the message receiver

**Fig. 3:**
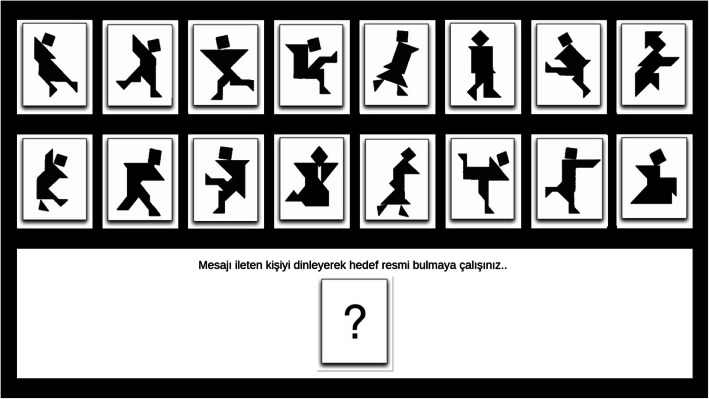
Message receiver’s screen: Instructions indicate that target image should be identified based on sender’s description

**Fig. 4:**
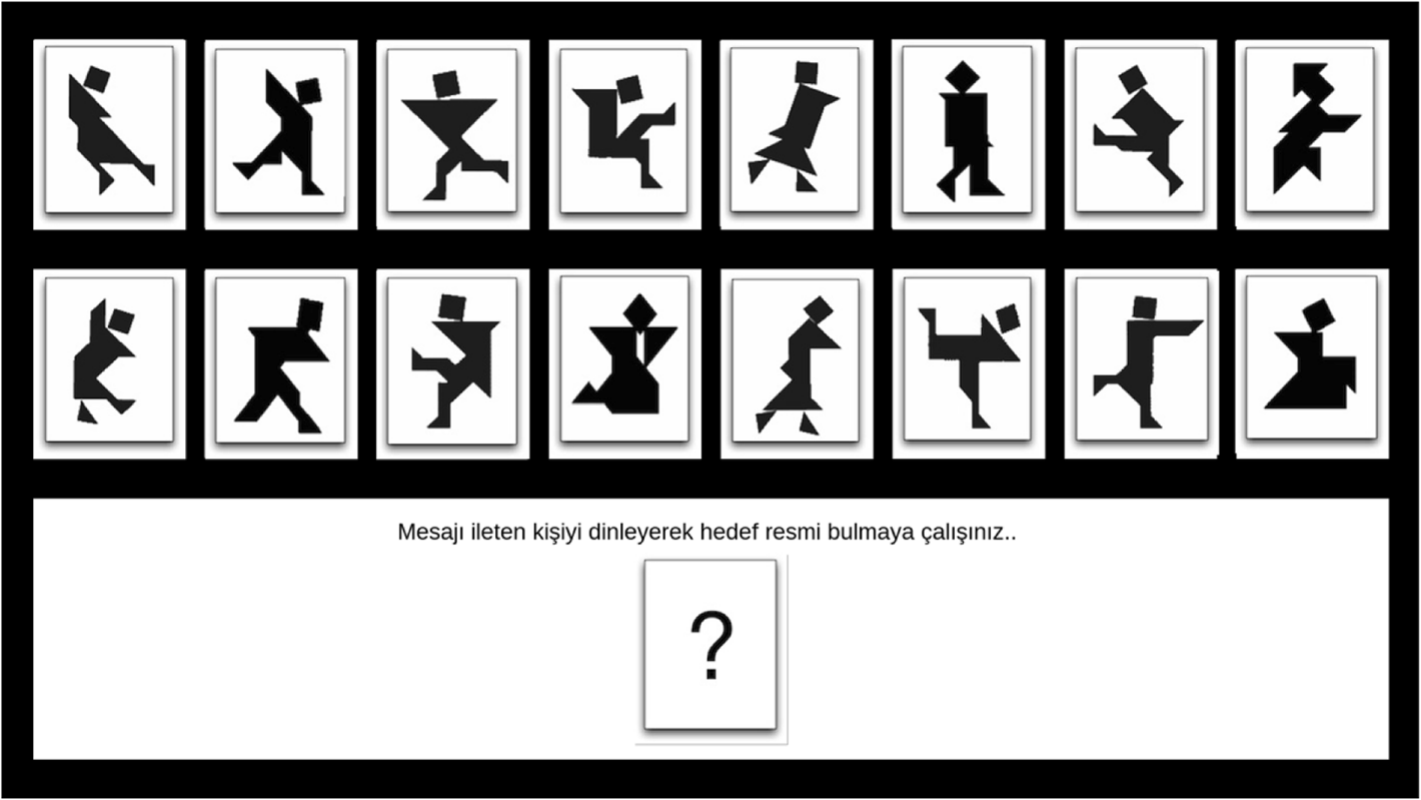
Bystander’s screen: Instructions indicate that participant should wait

Participants were brought into the lab in groups of three and seated randomly at terminals separated by partitions. After being instructed on the use of headsets, they were directed to turn their attention to their individual screens. Participants first saw an informed consent form. Upon giving their consent, they were shown a video describing the experimental game. The video explained the three roles they could be asked to play in each round of the game—message sender, message receiver, and bystander—and presented sample screens associated with each role (Figs. 2, 3, and 4). It also informed subjects that their payment would be contingent on the group’s overall performance and determined by the duration of the rounds and the number of mistakes made during the game. After watching the video, participants were presented with two questions testing their understanding of the roles and payment conditions. Those that made a mistake were shown the video again.

Each group of three played 120 rounds in the matching game. Role assignments—indicating which dyad would actively communicate—and the target image to be described for each round were randomly determined at the start of the game by the software program. While role assignments were randomly determined, role distributions throughout the game depended on the experimental configuration (030C or 030T) to which the game was randomly assigned. 030T groups played the game in dyads *ij*, *ik*, and *jk* in random order, while 030C groups played the game in dyads *ij*, *jk*, and *ki* in random order. The target image for each round was also randomly determined.

In each round, the participant assigned to the role of message sender tried to describe the target image for that round to the participant assigned to the role of message receiver. The message receiver tried to correctly identify the target image and select it on her/his screen. Message senders and receivers communicated freely through headsets. Senders also saw receivers’ guesses. Each round continued until the receiver identified the correct image. Participants’ screens varied with respect to the order in which the 16 tangrams were placed on the screen so that they could not give each other coordinates for the target images (Figs. 2, 3, and 4 do not reflect this as they show the sample screens shown to the participants in the instructional video). Each participant saw the tangrams in the same location in each round, however, so there would be no confusion arising from target placement across rounds.

Participants’ speakers and microphones were controlled by the experimental software so that only the message sender and receiver could speak during a round. Bystanders were allowed to hear the focal dyad and see the images picked by the message receiver in the “transparent communication” condition but not in the “private communication” condition. Bystanders in the private communication conditions heard a recording of rainfall so they could not hear the speaker or receiver. Bystanders were not allowed to speak in either condition.

The receiver was able to make more than one selection, and the dyadic interaction continued until the receiver selected the correct image on the screen. Once the correct selection was made, the experiment progressed to the next round. The experimental software recorded all selections of the receiver (providing the mistake count in the round) and the time spent until correct selection. Table 1 presents transcripts of several rounds of two different experimental sessions where subjects describe the same image.

**Table 1:** Selected rounds from two experimental sessions where the same image is described

Acyclic, transparent communication in a group consisting of friends:	
Round: 6 i->k Time: 65 seconds Mistake count: 2	S: Okay… I don’t think I can describe this. (Not audible). This also has a rhombus. It’s as if the person is standing, turned to the side and posing. Errr, how to..? Like a rhombus, the bottom part is like a triangle but it’s like a rocket going up. Think about it like that. Straight up. Imagine that beneath the square a parallel line is going up like a rocket. (Laughter) R: Gah! I can’t find it. Does it have, like, a hunched back? S: No, no hunch. Imagine a rocket. When you look at it, it’s more like a rocket than a person. R: The bottom part… Is it the upper body that’s a triangle? S: Yes, the upper body is a triangle but the lower part… R: Ok, got it. S: Okay.
Round: 11 i->k Time: 17 seconds Mistake count: 1	**S:** Right, I’m starting. Again, it has a head but it’s like a rocket. Imagine something going up like a rocket. **R:** Is it going left? **S:** It’s going left. So… **R:** Okay.
Round: 23 i->k Time: 9 seconds Mistake count: 0	**S:** Rocket. Imagine a rocket going up.
Round: 86 j->k Time: 5 seconds Mistake count: 0	**S:** Rocket.
Cyclic, transparent communication in group composed of friends and strangers:	
Round: 5 k->i Time: 33 seconds Mistake count: 0	**S:** In this picture, it’s like, the figure has a diagonal body. Both feet are in the air, she/ he looks as if she/he jumped while dancing, the head is inclined. **R:** Err, like, the head is inclined and like, not touching the neck. **S:** Yes... The body is like a diagonal. **R:** Hmm, yes. The feet should be to my right and the head should be to my left. **S:** Yes. **R:** Then it must be this one. **S:** Yes
Round: 19 k->i Time: 45 seconds Mistake count: 1	**S:** One foot is on the ground, one foot is in the air. The body is diagonal... **R:** Mm hmm… **S:** As if she/he is dancing. Like, as if it’s Michael Jackson. So like, the body is diagonal. The arms aren’t visible. **R:** Errr … **S:** His/her feet... **R:** Is there a backward protrusion from his/her waist? **S:** There is, yes. **R:** Is there a forward protrusion from the shoulder? **S:** Not really. **R:** The foot that’s on the ground isn’t flat, right? It’s diagonal… **S:** No, it’s not flat, it’s sort of like on its toes. **S:** No, that’s not the one. No, that’s not it. Both feet are close to the ground, only one is in the air. **R:** Is it the one with a diagonal body? The head… The head… **S:** Yes, the body is diagonal. **R:** The head doesn’t touch? **S:** That’s right, it doesn’t touch. **R:** Then this should be Michael Jackson.
Round: 26 k->i Time: 7 seconds Mistake count: 0	**S:** Michael Jackson... The diagonal one.
Round: 102 j->k Time: 6 seconds Mistake count: 0	**S:** Err, Michael Jackson.

Note: Third person pronouns are un-gendered in Turkish. We use she/he and her/his where the subjects used a third person pronoun

After 120 rounds of play as a triad, participants were asked to describe each image privately. Their verbal descriptions were recorded. Next, participants responded to questions about their age, gender, and how many of the other two participants they knew. In the subsequent screen, each participant received information about the group’s total number of mistakes, the average round completion time they achieved, and the payment per person. Payment was calculated according to the formula: 450/average round time – average round mistake × 10 for open communication groups, and 750/average round time – average round mistake × 10 for private communication groups, overriding if necessary to make sure no one got paid less than 20 or more than 35 TRY (which, at the time, corresponded to 7 and 12 USD). Finally, participants progressed to a debrief screen. After the subjects read the debrief screen and entered any comments they had, the administrator made the payment in cash, thanked the participants and concluded the experiment.

### Participants

The experiment was conducted in Istanbul Turkey, at Sabanci University’s School of Management and Istanbul Technical University’s (ITU) Entrepreneurship and Innovation Center (GINOVA). IRB approval was granted by both universities. Participants were recruited through fliers hung at both campuses, announcements in courses, and a banner on GINOVA’s website. All participants were paid 20 TL for participation in the experiment and up to 15 TL more for performance. Participants recruited through courses also received 5% course credit for their participation. (Other opportunities to receive equal credit were provided to students who chose not to participate.) About half of the experimental sessions had at least one participant who received course credit for participation.

The experiments were conducted in two phases^1^. Study 1 had 73 groups (21 private C, 18 C with transparent communication, 17 T private, 18 T with transparent communication). One private C group is missing code data because one of the participants did not record their descriptions. One of the private T groups is missing speed data due to computer malfunction.

In study 2, amendments were made to the software so that each participant was informed of the focal dyad in each game. This was done to avoid any possible confusion in T groups, where *i* could be speaking to *j* or *k*, and *k* could be hearing from *i* or *j*. Participants in study 2 were also told at the beginning of the experiment whether they would be playing in a T or C structure. We call this the “clear structure” condition. 11 C and 13 T groups (all with private communication) were run in study 2. We failed to record codes and speed for one of the C groups. Two sessions were cut short due to technical problems (ending after 50 and 104 rounds).

### Data coding and analysis

One of the authors and a research assistant coded transcriptions of the recordings that participants made of their labels for each image at the end of the experiment. Labels were recorded for 1515 images generated by 95 experimental groups (the missing 5 labels are due to participants skipping the first image without recording labels). Labels provided by participants in each group for each image were compared to one another and classified as including a common code at the group level, a single dyadic code, multiple dyadic codes, or no codes. Classification was done on a worksheet that did not include any identifying information about the experimental condition. Table 2 shows stylized examples of how codes were classified. The 1515 codes, classified into the categories shown in Table 2, constitute the data analyzed in Tables 3 and 4.

**Table 2:** Hypothetical example of how emergent codes were classified based on labels given by participants at the end of the experiment

Agent	No common code	Only dyadic codes	At least one group-level code	Single group code	Single group code w/ one word label
i	“superman”	“superman” + “Xmas tree”	“superman”	“Xmas tree”	“tree”
j	“Xmas tree”	“superman”	“superman” + “tree”	“Xmas tree”	“tree”
k	“Nepalese flag”	“Xmas tree”	“superman” + “tree”	“Xmas tree”	“tree”

**Table 3:** t-tests of correlation between communication effectiveness (speed and accuracy) in the last round of game played with a target image and the kind of code that was elicited at the end of the experiment for that target image

Test performed on set =>	All codes (*N* = 1515)			Some dyadic or group level code exists (*N* = 1389 for time, *N* = 1405 for accuracy)			At least one group level code exists (*N* = 846 for time, *N* = 853 for accuracy)			There is only one group code (*N* = 641 for time, *N* = 647 for accuracy)		
	Completely divergent labels	Some dyadic or group level code exists	Difference	Dyadic codes only	At least one group level code exists	Difference	There are multiple codes	There is only one group code	Difference	The label is longer than one word	The label is a single word	Difference
mean time to complete last round in seconds (st.dev in parentheses)	13.85 (8.61)	11.37 (10.44)	2.48**	13.2 (11.61)	10.2 (9.44)	3**	11.81 (8.35)	9.68 (9.71)	2.14**	9.78 (9.86)	6.73 (1.24)	3.06†
average number of mistakes made in last round (st. dev in parentheses)	0.15 (0.40)	0.08 (0.40)	0.06†	0.12 (0.55)	0.06 (0.27)	0.07**	0.07 (0.30)	0.05 (0.26)	0.02	0.05 (0.26)	0.05 (0.21)	0.01

Single-tailed, two-sample t-tests with equal variances

** *p* < 0.01; * *p* < 0.05; †*p* < 0.1

**Table 4:** t-tests of the probability of code development as a function of experimental condition

Panel 1	All groups (*N* = 1515)			All groups (*N* = 1515)			All groups (*N* = 1515)					
	Transparent com’n	Private com’n	Difference	Strangers	Not all strangers	Difference	Acyclic	Cyclic	Difference			
P (not completely divergent | entire set of codes)	0.95 (0.22)	0.92 (0.28)	0.03**	0.93 (0.26)	0.93 (0.26)	0	0.93 (0.26)	0.93 (0.26)	0			
P (at least one group code exists | not completely divergent)	0.74 (0.44)	0.52 (0.50)	0.21**	0.53 (0.50)	0.62 (0.49)	−0.09**	0.59 (0.49)	0.62 (0.49)	−0.03			
P (a single group code exists | some group code exists)	0.86 (0.35)	0.67 (0.50)	0.18**	0.71 (0.45)	0.76 (0.42)	−0.05	0.76 (0.43)	0.76 (0.43)	0			
P (a one-word label is used by entire group | a single group code)	0.05 (0.22)	0.01 (0.11)	0.04**	0.04 (0.20)	0.03 (0.18)	0.01	0.04 (0.19)	0.03 (0.17)	0.01			
Panel 2	All groups with private comm’n (*N* = 947)			All groups with transparent comm’n (*N* = 568)			All groups with private comm’n (*N* = 947)			All groups with transparent comm’n (*N* = 568)		
	Acyclic	Cyclic	Difference	Acyclic	Cyclic	Difference	Strangers	Not all strangers	Difference	Strangers	Not all strangers	Difference
P (not completely divergent | entire set of codes)	0.92 (0.28)	0.91 (0.27)	0.01	0.94 (0.24)	0.95 (0.21)	−0.01	0.93 (0.25)	0.91 (0.28)	0.02	0.92 (0.28)	0.95 (0.22)	−0.03
P (at least one group code exists | not completely divergent)	0.53 (0.50)	0.52 (0.50)	0	0.71 (0.46)	0.77 (0.42)	−0.07*	0.49 (0.50)	0.53 (0.50)	−0.04	0.64 (0.49)	0.75 (0.43)	−0.11*
P (a single group code exists | some group code exists)	0.67 (0.47)	0.67 (0.47)	0	0.86 (0.35)	0.85 (0.36)	0.01	0.63 (0.49)	0.68 (0.47)	−0.05	0.93 (0.26)	0.85 (0.36)	0.07
P (a one-word label is used by entire group | a single group code)	0.1 (0.11)	0.1 (0.11)	0	0.06 (0.24)	0.05 (0.21)	0.01	0.02 (0.15)	0.01 (0.11)	0.01	0.08 (0.27)	0.05 (0.22)	0.03
Panel 3	All acyclic groups (*N* = 755)			Groups composed of strangers (*N* = 207)			Acyclic groups with private comm’n (*N* = 470)			Groups composed of strangers with private comm’n (*N* = 159)		
	Strangers	Not all strangers	Difference	Acyclic	Cyclic	Difference	Strangers	Not all strangers	Difference	Acyclic	Cyclic	Difference
P (not completely divergent | entire set of codes)	0.93 (0.26)	0.93 (0.27)	0	0.93 (0.26)	0.92 (0.27)	0.01	0.94 (0.23)	0.91 (0.29)	0.03	0.94 (0.23)	0.88 (0.34)	0.07†
P (at least one group code exists | not completely divergent)	0.48 (0.50)	0.62 (0.49)	−0.14**	0.48 (0.50)	0.63 (0.49)	−0.15*	0.47 (0.50)	0.55 (0.50)	−0.08†	0.46 (0.50)	0.61 (0.50)	−0.14†
P (a single group code exists | some group code exists)	0.57 (0.50)	0.79 (0.41)	−0.21**	0.58 (0.50)	0.95 (0.23)	−0.37**	0.54 (0.50)	0.72 (0.45)	−0.18**	0.54 (0.50)	0.94 (0.24)	−0.41**
P (a one-word label is used by entire group | a single group code)	0.05 (0.23)	0.04 (0.19)	0.01	0.05 (0.20)	0.03 (0.17)	0.03	0 (0)	0.02 (0.13)	−0.02	0 (0)	0.06 (0.25)	−0.06†
Panel 4	All groups with private comm’n (*N* = 947)			Acyclic groups with private comm’n (*N* = 470)			Acyclic groups composed of strangers with private comm’n (*N* = 127)					
	Clear structure	Unclear structure	Difference	Clear structure	Unclear structure	Difference	Clear structure	Unclear structure	Difference			
P (not completely divergent | entire set of codes)	0.88 (0.32)	0.93 (0.25)	−0.05**	0.91 (0.28)	0.92 (0.27)	−0.01	0.98 (0.15)	0.93 (0.27)	0.05			
P (at least one group code exists | not completely divergent)	0.51 (0.50)	0.53 (0.50)	−0.02	0.53 (0.50)	0.52 (0.50)	0.01	0.46 (0.50)	0.47 (0.50)	−0.01			
P (a single group code exists | some group code exists)	0.65 (0.48)	0.68 (0.47)	−0.03	0.66 (0.48)	0.69 (0.47)	−0.03	0.48 (0.51)	0.57 (0.50)	−0.09			
P (a one-word label is used by entire group | a single group code)	0 (0)	0.02 (0.14)	−0.02†	0 (0)	0.02 (0.15)	−0.02	0 (0)	0 (0)	0			

Standard deviations are in parentheses

Single-tailed, two-sample t-tests with equal variances; ** *p* < 0.01; * *p* < 0.05; †*p* < 0.1

We test the effectiveness of each type of code through *t* tests of the speed and accuracy of the last round that a triad played with an image (Table 3).

We also use *t* tests to test the causal effect of the experimental conditions on the probability that a particular type of code emerges in a triad (Table 4).

Tables 6 and 7 report multilevel mixed-effects linear regressions on round completion times that were performed on Stata 14 (StataCorp 2015, command “mixed”). These random intercept models estimate variation around the intercepts for experimental groups. Fixed effects regressions produce the same pattern of covariate estimates and are available upon request.

## Results

### Effectiveness of emergent codes

We start our analyses with a correlational test of the effectiveness of different kinds of codes for communication and coordination. Our goal is to provide a preliminary validation of the assumption that group-level codes are more effective than multiple specialized codes. Table 3 tests whether the speed and accuracy of a group during the last round that it played with a particular target image is correlated with the kind of code that was elicited for that image after the games ended. This is a conservative test for two reasons. First, the games are played by dyads, whereas the kind of code refers to the code at the triadic level. Second, we elicited participants’ preferred labels at the end of the experiment, by asking them to say how they would describe the image. It is possible that the participants would be able to comprehend the different labels of the other participants in their groups but did not report all of the labels they associated with each image.

Going from left to right in the table, we first examine the 1515 triad-target level codes for which we were able to measure the type of code that existed. Of these, 110 were completely divergent, that is, each of the three members of the group recorded a different label for the same image at the end of the experiment. The average time to complete a round was about 13.85 s for groups that offered completely divergent labels as compared to 11.37 s for groups in which at least two participants offered the same label at the end of the experiment. The average number of mistakes made in the former was 0.15, compared to 0.08 for the latter. The differences are statistically significant.

The second column compares speed and accuracy for triad-target pairs for which all three participants in the triad offered at least one common label or descriptor to targets for which there were some common labels or descriptors but none at the triad level. (That is, this sample excludes the 110 completely divergent codes.) We see that the existence of a group-level code, compared to only dyadic codes, improved both speed and accuracy of coordination. The differences are statistically significant.

The third column focuses on the sample of targets for which at least one group level common code exists. Some of these targets were given multiple labels, shared at the dyad or triad level. We test whether the presence of a single triadic code was correlated with speed and accuracy. We find a significant correlation with speed but not with accuracy. The existence of multiple codes made dyads 2 s slower on average.

The final column tests whether, among targets that were given a single group-level code by all three participants, labels that consisted of a single word were more efficient. The difference of 3 s is marginally significant (and there is no difference in accuracy).

Overall, this correlational analysis demonstrates that types of codes (whether dyadic or triadic, redundant or non-redundant, short or long) are associated with performance on coordination tasks. Communication is faster when codes are shared at the triad level, are non-redundant, or the labels are shorter. It is more accurate when codes are shared at the triad level. This confirms the prediction of formal models, which claim that codes shared at the group level are more effective, even for distributed coordination tasks (Cremer et al. 2007).

### Antecedents of emergent codes

Next, we test whether the emergence of codes with these characteristics can be explained by transparency versus privacy of communication, role differentiation in the communication structure, and prior common ground that existed among the participants. We present the results in four panels.

Table 4 tests the impact of each covariate on the probability of emergence of each type of code. The first panel shows the main effects of transparency of communication, absence of prior common ground, and acyclicity of communication structure. Tests in the first column indicate that groups engaged in transparent communication (where bystanders heard the conversation of the focal dyad), compared to those in the private communication condition, were significantly more likely to develop codes that we found in the above analyses to be more effective. In the first row, we see that their codes were less likely to be completely divergent. In the second row, we see that conditional on not being completely divergent, they were more likely to report codes shared at the triad level. In the third row, we see that given that they had some triadic code, they were also more likely to converge on a single triadic code. Finally, in the fourth row, we find that the single-triad-level code was more likely to be a single word among groups that had transparent communication. To summarize, private communication hurt groups’ ability to develop effective codes relative to transparent communication.

In the second column of Table 4's first panel, we perform similar tests comparing groups composed of all strangers and groups that were not composed of all strangers. We find that the former are less likely than the latter to have a group-level code but there is no other difference. In the last column of Table 4's first panel, we compare codes that emerged in acyclic and cyclic groups and find no statistical difference. These results indicate that communication structure or prior common ground do not have significant main effects. Nonetheless, given our prediction that transparency/privacy of communication may have moderating effects on communication structure and prior common ground, we perform separate *t* tests for private and transparent communication groups in Table 4. We do not find statistically significant differences between cyclic and acyclic groups (column 1) or between groups with and without prior common ground (column 3) in private communication groups. In fact, groups with transparent communication appear to have had greater difficulties with dyadic code harmonization in acyclic structures (column 2) and when lacking prior common ground (column 4).

Given our conjecture that prior common ground pertains not only to a shared lexicon but also a shared understanding of behavioral norms, we also investigate whether lack of prior common ground may inhibit code convergence in certain structures more than others. The second panel in Table 4 reports interaction analyses for communication structure and existence of prior common ground. In columns 1 and 2, we see that groups of strangers develop fewer effective codes than groups of non-strangers within acyclic groups (column 1) and acyclic groups of strangers develop fewer effective codes than cyclic groups of strangers (column 2).

Given the finding that private communication makes code convergence harder, we repeat these interaction tests for the subset of groups with private communication in the third panel of Table 4. Column 3 shows the same results as in column 1: groups of strangers are less likely than groups of non-strangers to develop effective codes when they are placed in acylic groups with private communication. Column 4 shows mostly the same results as in column 2, with one exception. We find that groups in the private communication condition that were composed of strangers were more likely to develop some code, that is, less likely to report completely divergent codes (row 1). However, conditional on developing some code, they were less likely to develop triad-level codes (row 2). Conditional on having at least one triad-level code, they were also less likely to converge on a single label (row 3). Given that they had a single label, this was less likely to be a single word (row 4) 2.

Can the difference between acyclic and cyclic groups composed of strangers be attributed to their confusion about their interaction partners? In study 2, groups communicated privately but were informed about the communication structure before the experiment and were notified of whom they were communicating with in each round. The final panel in Table 4 reports tests that separate private communication groups by clear versus unclear structure (that is, comparing study 1 and study 2). We find little difference between study 1 and study 2 in private groups overall (column 1), no difference in private groups with acyclic structure (column 2), or in private groups with acyclic structure that were composed of strangers (column 3). This suggests that the effect of communication structure cannot be explained by participants’ confusion about whom they were communicating with or their lack of knowledge about the communication structure.

In summary, it is the transparency of communication that has the strongest effect on emergence of codes. Structure of communication (role differentiation) does not have an effect on its own, but only in combination with lack of prior common ground. Convergence of codes into a group-level code is more difficult to achieve in acyclic structures when groups are composed of strangers. The question arising is thus: why is common ground harder to create in such cases?

One possible answer is that acyclicity creates difficulties in harmonizing dyadic codes into group-level codes and that strangers have a harder time overcoming these difficulties, compared to individuals with prior common ground. As noted earlier, common ground has multiple aspects. One is lexical—shared assumptions about how the lexicon refers to entities in the world. In our experiment, it is highly implausible that friends already have a common lexicon for the images we ask them to describe. While groups of friends may share experiences (such as a recent movie or an acquaintance) that help them come up with names for the novel images they see, this does not seem to help them much in our experiment: Groups of friends do no better than other groups at the start of the experiment or in cyclic conditions.

We think that what accounts for the difference between friends and strangers is likely to be a kind of behavioral common ground. By behavioral common ground, we refer to a broader set of conventions and possibly tacit shared understandings of how to behave in a communication situation: what “rules of conversation” or “ground rules” hold? If there is a conflict between codes, how do we resolve it? Lack of such behavioral common ground, which will increase “strategic uncertainty” in coordination tasks (Crawford 2017), may make it harder for individuals to agree on a common code. Instead, the group may accumulate dyadic codes, with individuals switching codes between the different dyads.^3^ We attempt to shed light on this conjecture in the next section.

### Process of code emergence

We attempt to get a closer look at how participants build common codes and why acyclic structures make this harder for strangers by analyzing the data on speed of coordination. Table 5 summarizes the variables we use in regression analyses. Tables 6 and 7 present estimates from regression models of the time it takes groups to complete each round of game. We see that groups composed of strangers take longer on average than other groups. Transparent communication increases the speed of communicative coordination (i.e., reduces the time to complete a round). Acyclicity of the triadic structure and clarity of the structure (whether participants were informed about the structure, that is, being in study 2) have no main effects.

**Table 5:** Summary statistics and correlations for variables used in the analysis of round-level data on coordination (*N* = 11,434)

Variables	Mean	St.D.	Min	Max	(1)	(2)	(3)	(4)	(5)	(6)	(7)	(8)
(1) Time to complete round (seconds)	19.82	24	4	528	1.00							
(2) Group composed of strangers	0.15	0.35	0	1	0.05	1.00						
(3) Transparent communication	0.37	0.48	0	1	−0.09	−0.13	1.00					
(4) Acyclic triad	0.49	0.5	0	1	0.03	0.13	0.02	1.00				
(5) Clear structure (Study 2)	0.24	0.43	0	1	0.03	0.04	−0.43	0.08	1.00			
(6) Round number	60.27	34.62	1	120	−0.38	0.00	−0.01	0.00	0.00	1.00		
(7) Cum. experience of dyad with target	1.24	1.33	0	9	−0.29	−0.00	−0.02	0.01	−0.01	0.55	1.00	
(8) Cum. experience of other dyads with target	2.47	2.1	0	12	−0.28	0.01	−0.01	0.00	−0.00	0.69	0.36	1.00
(9) Cum. experience of triad with other targets	56.57	32.5	1	119	−0.37	0.00	−0.01	0.00	0.00	1.00	0.52	0.65

**Table 6:**
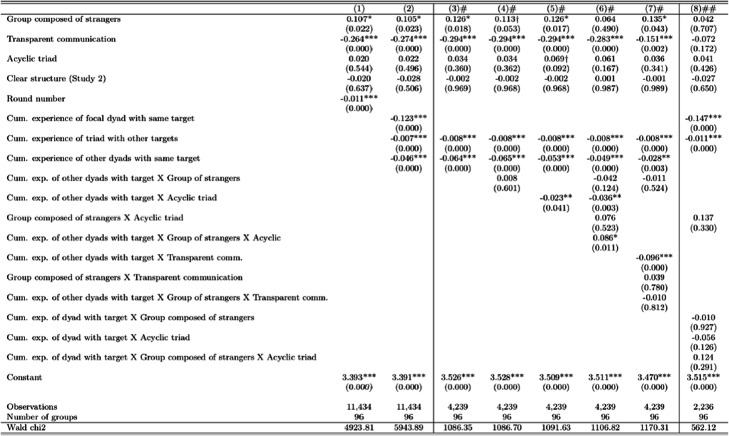
Coefficient estimates from mixed effects models of the time taken to complete each round of game (ln(seconds))

*p* values in parenthesis; *** *p*<0.001, ** *p*<0.01, *p<0.05, †*p*<0.1

# Sample of observations where focal dyad has no prior experience with target

## Sample of observations where other dyads have no prior expience with target

**Table 7:**
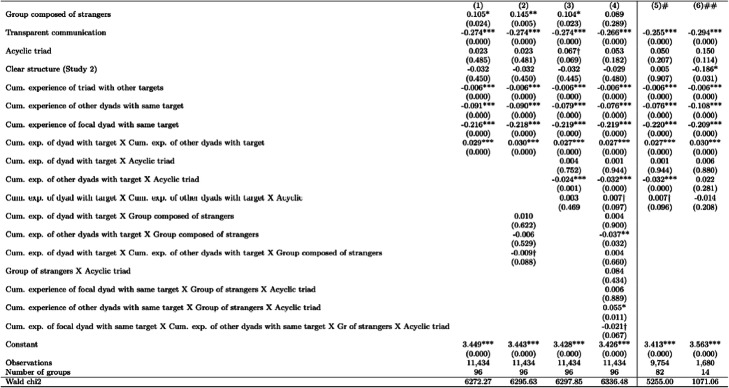
Coefficient estimates from mixed effects models of the time taken to complete each round of game (ln(seconds))

*p* values in parenthesis; *** *p*<0.001, ** *p*<0.01, *p<0.05,†*p*<0.1

# Groups not composed entirely of strangers

## Grooups composed of strangers

Our analysis focuses on triads learning from experience. The triad’s experience is proxied by round number in model 1 of Table 6. We see that groups coordinate faster as they progress through rounds of the game. In model 2, we separate triad’s prior experience into experience of focal dyad with the same target, experience of other dyads with the same target, and the triad’s experience with all other targets. The model shows that each of these components of experience contributes to learning, increasing the speed of coordination. Dyads learn most from their own experience with the same target. Other dyads’ experience also helps, showing that either senders or receivers are able to transfer some learning from their prior interaction with their other partner. The effect of the triad’s experience with other target images likely reflects learning about the game and the improvement in coordination gained from learning to distinguish images from one another.

We can explore a series of questions related to early experience by focusing on what happens when a dyad has no previous experience of a given target picture. Models 3 through 7 in Table 6 focus on the very first time that a focal dyad encounters a target image, by examining this sub-sample of observations. In model 3, coefficient estimate for the other dyads’ experience with the same target is again negative, showing that focal dyads with no experience of a target image benefit from other dyads’ experience—there is transfer of linguistic coordination. Model 4 shows that this transfer is no greater or lesser for groups composed of strangers. Model 5 shows that acyclicity helps dyads with no prior experience of a target learn from other dyads that have seen the target before. That is, acyclicity helps transfer of conceptual pacts from one dyad to another. Model 6, interacting acyclicity with lack of prior common ground, finds a significant slowing down of dyads that are faced with the task of describing a target for the first time after another dyad if they are comprised of strangers and are in an acyclic configuration. Including this interaction term also makes the main effect of lack of prior common ground become insignificant, suggesting that it is such situations of code conflict that accounted for the liability of coordinating with strangers. Model 7 tests the moderating effect of transparent communication and finds that it increases learning from the other dyads’ experience, but the effect is not different for groups composed of strangers.

Finally, model 8 performs a similar test on the converse situation—when the other dyads have no experience with the focal target. In this case, lack of prior common ground, acyclicity, or transparent communication have no impact on the speed of coordination. Dyads that have seen the target before benefit from their prior experience. However, this effect is not different for groups with prior common ground or groups with acyclic structures. This pattern of results confirms that acyclicity and lack of prior common ground impact coordination in situations of code conflict and do not have effects on purely dyadic learning.

So far, we have focused on dyads lacking experience with a particular target image and found that dyads in acyclic triads can learn more from the experience of other dyads unless the group is composed of strangers. In Table 7, we ask what happens when, as experience cumulates, both the focal dyad and the other dyads develop some mutual coordination on a given target. We expect a potential conflict arising from different dyadic conceptual pacts. Participants are likely to get confused about which labels they used with which partner. Individuals may try to consolidate labels across partners, harmonize dyadic codes, but meet resistance from their partners who have internalized other codes. Model 1 in Table 7 interacts the focal dyad’s experience and the other dyads’ experience with the same target and finds that the coefficient estimate is positive, indicating a slowing down of coordination. This effect illustrates the coordination difficulty that arises when dyads have developed dyad-specific conceptual pacts. The grounding that was accomplished in other dyads conflicts with that of the focal one, even though in each round, one of the participants in the dyads is the same. Models 2 through 4 show that dyads in acyclic groups learn more from other dyads’ experience. Dyads in acyclic groups composed of strangers, however, learn less from other dyads’ experience and also experience marginally lower confusion from conflicting codes. Models 5 and 6 split the sample by lack of prior common ground and further show that dyads in groups not entirely composed of strangers learn better from other dyads in acyclic relative to cyclic structures, while acyclicity does not improve learning from other dyads in groups composed of strangers.

In summary, our analysis of speed of coordination reveals more information on the process of dyadic code formation and its harmonization (or not) at the triad level. Acyclicity helps dyads learn from other dyads and on its own does not increase the conflict that arises from multiple dyadic codes. Dyads in groups composed of strangers, however, do not appear to benefit from acyclicity. Instead, they learn significantly less from other dyads when placed in acyclic configurations. Combined with our analysis of the emergent codes above in Table 4, these results show that groups of strangers in acyclic triads retain different codes in each dyadic relationship rather than harmonize them into one common code. They form and use multilingualism (with attendant code-switching) to accommodate conflicting codes in their dyads. We think the reason this happens is that individuals with no prior interaction find it more difficult to pursue a group-level code within a differentiated role structure (where one agent (*i*) is only a sender, and another (*k*) is only a receiver) despite the less differentiated relational structure (whereby *i* (*k*) could be sending to (receiving from) either of two dyadic partners).

## Discussion

An important result of our experiment is that a multiplicity of coexisting codes may arise within a triad. Even though multiple decentralized dyadic codes are less efficient than group-level codes, they are routinely created in distributed coordination tasks and stabilize as an equilibrium through such interactions. Economic and managerial perspectives may explain this by the fact that unlearning (some of) the dyadic codes in order to create group-level codes, or otherwise harmonizing dyadic codes into an emergent group-level code, is more costly than using specialized dyadic codes (Wernerfelt 2004; Cremer et al. 2007). However, this cannot explain the persistence of dyadic codes in our experiment, because the advantages of having specialized dialects versus convergent group-level codes did not vary across experimental conditions. Instead, our results seem to provide evidence of the Simmelian argument that triads may disintegrate into three parties of two persons each. We show that the way in which dyads are configured into a triad and the prior history of the individuals composing the group affect the likelihood of this happening.

In other words, coordination difficulties that arise from structure are social, not technical, in nature. They arise from second-order questions around how to coordinate. The behavioral common ground that is required in our experimental game involves both dyadic and triadic coordination. Within dyads, individuals need to agree on what the roles of sending and receiving messages imply—such as who, if any, should adopt the other’s preferred labels. Prior work finds that dyads are sometimes able to solve such relational coordination problems by adopting an asymmetric imitation routine (Selten and Warglien 2007). Within triads, distributed dyadic coordination presents additional requirements for behavioral common ground. Interacting independently of each other, each dyad forms its own conceptual pacts. Not only do triadic structures create greater challenges of lexical coordination, but they also may make it harder for individuals to devise “ground rules” for resolving conflicts among conceptual pacts and harmonizing dyadic codes into triadic ones.

Our data on coordination times suggests that dyads in acyclic triads are better able to learn from the other dyads’ experience. Acyclicity helps the transfer of conceptual pacts from one dyad to another. This may be due to two individuals in the triad specializing in sender or receiver roles and harmonizing code development with each of their two partners. However, our findings also indicate that navigating acyclic structures to achieve gains in coordination requires behavioral common ground. Role differentiation and asymmetry in the acyclic triadic structure poses greater “strategic uncertainty” for individuals in these groups. This is evidenced by dyads in groups composed of strangers, who do not appear to benefit more from other dyads’ experience when they are placed in acyclic structures. These groups develop and retain different codes in each dyadic relationship rather than harmonize them into one common code. Acyclicity creates Simmelian breakdowns for strangers, while it aids global convergence for groups with prior common ground.

Why are individuals with prior common ground better able to navigate acyclic structures and benefit from their structural advantages? Why do individuals with no prior interaction find it more difficult to pursue a group-level code within a differentiated role structure? Supplemental analyses suggest that friends make more mistakes at the beginning of the experiment than strangers. Strategic uncertainty among strangers makes them more cautious, leading to slower communication but no greater errors. The effort to avoid errors leads to generation of new conceptual pacts within each dyad and thus an accumulation of multiple labels and longer codes. It is possible that strangers are stalled more by strategic uncertainty than friends are, presumably because they have a greater fear of making mistakes. They may suffer from a deficit of “social fluency,” requiring a longer process of acquiring a common understanding of the differentiated behavior that is expected from each of them in interaction. They need to learn how their actions, and others’ actions contingent on their actions, are appropriate.

Our study suffers from a few notable limitations. One concerns the triadic configurations we have elected to compare. All our groups were composed of three asymmetric dyads. Extending the framework beyond asymmetric triads, it would be useful to also compare these to a triad composed of three mutual ties, with all individuals playing both sender and receiver roles within each dyad. Another useful comparison would be a triad with one sender and two receivers who do not interact with each other—a configuration that forms the building block of branching hierarchies. Further insights about the effects of interactional structures may be gained from comparisons of different acyclic structures. Role differentiation in 030T necessarily implies that two agents (*i* and *k*) perform their roles of sender or receiver with two other agents, whereas each agent in 030C always sends messages to one person and receives messages from a different person. This might make it easier for agents (in particular strangers perhaps) in 030C triads to manage emerging conceptual pacts. Future experiments may be designed to disentangle specialization of roles at the dyadic level from the non-specialization of roles at the triadic level.

A second important limitation concerns the fact that we did not collect data on codes as they evolved during the experiment. Analyzing entire transcripts of code evolution would have allowed us to directly observe individuals experiencing conflicts between dyadic codes and attempting to overcome them. It is possible that the methods by which people achieve common understanding (e.g., conversational rules or the use of deferential roles) vary across different structures. Future work may use conversation analysis to examine whether the difference in T and C structures gives rise to differences in the interactions leading to mutual understanding (Garfinkel 1967; Sacks 1992).

Another useful addition to future experiments of this type would be to assess the extent of balkanization, or discord between individual members of triads, by collecting measures of conflict (De Wit et al. 2012). These may give some insight into the process and difficulties of establishing behavioral common ground across different communication structures.

## Conclusion

Organizational economics illuminates the inherent trade-offs associated with code specialization, the sociological perspective invites us to look at the relational context of code creation, and psycholinguistics provides the tools for studying the process of creating common codes in groups. Our study builds on prior work in these fields to make fruitful connections to organization studies. It thus provides a partial answer to the call for an “organizational linguistics” (Brandenburger and Vinokurova 2012). Perhaps, the most important contribution of the present study is to indicate future avenues of research that continues to build these connections. One such direction is the study of behavioral common ground. What do participants in organizations need to know in order to integrate their efforts with the contributions of others?

Our finding that acyclicity causes friction in groups composed of strangers is surprising in light of prior work showing human proclivity to form (Carnabuci et al. 2018) and process hierarchical relationships (Zitek and Tiedens 2012). Our results show that acyclicity in structure is by itself not sufficient to cue strangers into a shared definition of the situation. Instead, it appears that a “cultural ordering schema” (Ridgeway 2006) may be necessary for individuals to figure out how to navigate acyclic structures and benefit from their potential for coordination when the structure does not impose a clear coordination logic (Argote et al. 2018).

Our study also has practical implications for organizations, especially those that engage in distributed work, as many had to do during emergency situations such as Hurricane Katrina or the Covid-19 pandemic. We find that even transient lack of coordination across groups that engage in distributed communication leads to the emergent code being less efficient. This suggests that organizations that want their members to share the same code should consider ways to make distributed communications more fluent. Our findings show that two interventions may help: making communications more transparent and instituting “ground rules” for communicative interactions. Organizations can make communications more transparent by ensuring that everyone concerned is on the same communication thread and has access to an archive of all prior exchange, such as by employing messaging platforms like Slack or Microsoft Teams. Our experiments show that such tools should be useful even when organizations are no longer required to ask their employees to work from home.

To establish ground rules for working in balkanized structures, organizations can institute formal ranks and titles (e.g., a designated “coordinator” who summarizes decisions at the end of a distributed discussion) or develop shared behavioral norms. Our study shows that having clear ground rules is especially important when communication structures are acyclic and that establishing trust among individuals so that they are not afraid to make mistakes in interaction will help harness the efficiency of transitivity.

More broadly, our study suggests that convergence on codes within organizational fields and markets likely depends not only on how often individuals interact and how they relate to one another (e.g., Koçak et al. 2014; Centola and Baronchelli 2015), but also how they coordinate across those interactions.

## Data Availability

The datasets generated and analyzed in this study are not publicly available due to commitments of confidentiality made to TUBITAK, but are available from the first author on reasonable request.

## Footnotes

1 Data collected in an initial phase are not used in this paper. We realized while running the initial 27 experimental groups that some bystanders were able to hear their group members despite the use of soundproofed headsets, depending on how loud the sender and receiver spoke. This effectively turned some of the intended private communication into transparent communication but not for all dyads or triads, contaminating the code data for the purposes of analyses conducted in this paper. We use only the data that was collected after a revision of the software so that a continuous recording of pouring rain was played over the headset of the bystander in the private communication condition.

2 Only three experimental groups were composed of strangers and placed in the transparent communication condition. This prevents us from testing the three-way interaction effect of lack of prior common ground X transparent communication X acyclicity on the effectiveness of emergent codes. Analyses of the time it takes groups to coordinate do not indicate that transparency of communication moderates the effect of either communication structure or prior common ground.

3 A similar instance of label accretion has been reported by Christie et al. (1952), Macy et al. (1953), who find that groups that face signal ambiguity or noise reduce errors by producing redundant codes, that is, synonyms. They trace the causes of noise and subsequent redundancy to lack of feedback on errors, symmetric links, and differences in positions. When subjects realize that they are making errors (thanks to feedback), they try to correct them by developing redundant codes (thanks to symmetric links). But this is harder to do when all communication has to pass through a central person as in the star network (differences in positions). While we observe a similar outcome, their explanations are not applicable to our experiment. In our groups, communication is always dyadic (e.g., communication between *j* and *k* does not pass through *i*), ruling out the positional explanation. We also have both feedback and two-way communication in both C and T.
